# The Therapeutic Goals Set by University Students in an Anonymous Web-Based Therapy and Support Setting

**DOI:** 10.3389/fpsyg.2022.745537

**Published:** 2022-08-08

**Authors:** Terry Hanley, Julie Prescott, Aaron Sefi

**Affiliations:** ^1^Manchester Institute of Education, University of Manchester, Manchester, United Kingdom; ^2^Department of Psychology, School of Education and Psychology, University of Bolton, Bolton, United Kingdom; ^3^Kooth, London, United Kingdom

**Keywords:** web-based therapy, therapy goals, goal-based outcome measure, higher education, mental health-state of emotional, social well-being

## Abstract

The interest in student mental health and wellbeing has increased in recent years. Additionally, there is a rising volume of students seeking support. Numerous online resources have been developed to meet this need, including anonymous web-based therapy. To date, there has been little focus upon how students may utilise such a service, and this study examines routine evaluation data (solicited from a Goal-based Outcome Measure) from a United Kingdom based service provider. Over the course of one academic year (2018–2019), 211 students articulated therapeutic goals within *Kooth Student*, a web-based therapy and support service for individuals in higher education. These goals were examined for key trends. The students identified a total of 625 goals to work on in therapy, with individuals setting an average of three goals each. The most common goals focused upon obtaining additional support within the service and exploring their emotions. The results suggested that female students were more likely to move towards achieving their goals, with goals that did move shifting an average of 7.74 on a 10-point scale. Practical goals that focused upon getting more help, both inside and outside the service, were most likely to be achieved. In contrast, self-help/self-care goals were less likely to be achieved. These results provide a helpful insight into how students made use of therapy and highlight the importance of the interaction that web-based services have with other provision (web-based and in-person). They also demonstrate the challenge of capturing meaningful outcome data in anonymous services.

## Introduction

In recent years, there has been an increasing focus on student mental health and wellbeing (Hubble and Bolton, 2019). Linked to this, within countries such as the United Kingdom (UK), a number of reports suggest that there has been a decline in the mental health and wellbeing of the student population (Thorley, 2017; Hubble and Bolton, 2019). This appears to have worsened during the COVID-19 Global pandemic, with numerous reports indicating that mental health and wellbeing difficulties appear to have increased in student populations during this difficult period of time (Burns et al., 2020). As the impact of mental health issues on students can be serious and lead to academic failure, dropping out of education, poorer career prospects and, in the worst cases, suicide (Hughes and Spanner, 2019), considering the ways in which students can be best supported is high on the agenda. This paper therefore explores the growing provision of web-based therapy to contribute to meeting this need.

### The Growth of Web-Based Therapy and Support in Higher Education

Since the 1990s, there has been a steady growth of web-based therapy and support services. These services offer support to individuals and groups in a variety of media, including text-based asynchronous communication (email) and synchronous communication (instant messaging), video conferencing support and self-administered Internet-delivered systems (SAID) of support (Barak et al., 2009). These services have become popular and, although pose many ethical and practical challenges, can arguably meet demands that traditional in-person services cannot always meet (Stoll et al., 2020). For instance, it is notable that web-based services are more appealing for some younger people (Ersahin and Hanley, 2017) and student populations (Hanley and Wyatt, 2021), the Internet making the access of services easier and increasing the anonymity of individuals while using them. Such benefits have particularly come to the fore during the recent pandemic, with web-based services reporting large increases in their service usage as a consequence of national policies such as school closures and social distancing measures (Inkster and Digital Mental Health Data Insights Group, 2021).

Higher education (HE) institutions have started offering a wide variety of services online. These include static resources that provide information and more dynamic resources, such as online therapy (Hanley, 2020). As noted above, web-based services prove a popular idea with student populations (Hanley and Wyatt, 2021). Despite this, research also suggests that engagement with such services might be relatively limited (Musiat et al., 2014; Santucci et al., 2014). For instance, within a study evaluating university implemented computerised cognitive behavioural therapy (CBT), 88% of participants (*n* = 43) did not complete the course of eight sessions (Santucci et al., 2014). High levels of attrition, 61.7% of participants (n = 1,141), were also reported in Musiat et al. (2014) paper investigating the efficacy for HE students of a CBT informed transdiagnostic online intervention. There seems to be limited research however that considers how students are engaging and experiencing web-based therapy directly offered by professionals or why levels of attrition appear to be high for web-based support.

### Goals in Web-Based Therapy

Self-direction and goals have long been important within therapeutic work (Hanley et al., 2015). Goals have been aligned to theoretical positions that prize individuals having choice in the direction that therapeutic work may head (Cooper and McLeod, 2011) and they have been associated with positive outcomes (Norcross and Wampold, 2018). Further, it is argued that this way of working brings to fore the voice and wishes of younger services users, a factor that has historically been neglected, and increases engagement with therapeutic services (Jacob et al., 2017). Thus, there have been moves to orientate therapeutic work with young people and young adults around goals (Hanley, 2012; Law and Jacob, 2015). In recent years, goal-based outcome measures (GbOM) have been deployed across many Child and Adolescent Mental Health Services (CAMHS) across the UK. Evaluations highlight that these idiographic measures prove more capable than standardised equivalents at capturing relevant change (Edbrooke-Childs et al., 2015) and are now used to indicate the preferential standardised measure for CYP (Jacob et al., 2017). To date however, there has been no systematic research focusing upon goal-focused work with young adults in HE settings.

Given the complex nature of online therapeutic services for younger individuals, using standardised outcome measures has proven both challenging and problematic (Sefi and Hanley, 2012). Much of the challenge has been associated to the specific benefits associated with working in this medium noted above. In particular, the anonymous nature of such contact enhances the complexity of interpreting outcome scores. Idiographic measures, such as GbOM, have therefore been suggested as one means of capturing appropriate evaluative information and increasing engagement in services (Sefi and Hanley, 2012; Jacob et al., 2021). In considering this further, Ersahin (2016) developed a taxonomy of goal categories based upon the goals that young individuals set in web-based therapy sessions. These included goals that were ‘intrapersonal goals’, ‘interpersonal goals’ and ‘goals on self, related to others’. This taxonomy provides a helpful way a categorising the goals that individuals articulate in online therapy. When compared to in-person therapy, it was notable that the goals generated online appeared to support individuals to access additional support and to address goals that may have been more intimate in nature (Hanley et al., 2017). It is unknown if similar trends are evident in work with students in HE.

### Rationale and Research Question

The move to offer web-based mental health and wellbeing support to students needs further scrutiny. While such services provide numerous opportunities, they also highlight many challenges. This brief report intends to contribute to the debates in this arena by examining the types of goals that students articulate when engaging with web-based therapy services. Further, it reflects upon the progress made towards these goals by those seeking support. The following three research questions are considered as,

1. What is the demographic profile of the students who use a web-based therapy service?
2. What categories/types of goals do students set while attending web-based therapy?
3. How do students’ progress with goals that are set during a period of web-based therapy?

## Materials and Methods

This is a practice-based research project (Holmqvist et al., 2015) using anonymized routine evaluation data from the *Kooth Student* web-based therapy and support service. *Kooth Student* is commissioned by HE providers and free at the point of delivery for those who access it. It allows individuals to obtain anonymous support and is completely text-based. While the service is multifaceted, and offers a variety of support pathways, this project focuses solely upon its web-based therapy resource in which trained professionals offer therapy to those seeking support.

The project was granted a favourable ethical review by the institution of the second author.

### Participants

During the period of review, notably the 2018/2019 academic year, the *Kooth Student* resource was available to all students at one HE institution within the UK. All the students that made use of the service and articulated goals were included in this study.

In total, 211 students articulated goals during the 12-month period. Demographic information about these individuals is presented at the start of the results section. These students identified 625 goals using the web-based Counselling Goals System (CoGS) embedded within the service. The CoGs follows the same format as pen and paper GbOM (e.g. Law and Jacob, 2015) by requesting individuals to self-define the goals that they currently have. It encourages individuals to identify whether their goals are ‘immediate goals’, ‘therapy goals’ or broader ‘life goals’. Further, the CoGS was developed so that it is visually attractive and easy to complete online.

### Data Generation: Goal Articulation and Routine Goal Monitoring

All individuals who accessed the *Kooth Student* service during the 12-month period were included in the study. This included individuals who organised pre-booked sessions with therapists, and those who use the more *ad-hoc* drop-in service that is offered. During the first contact with the service, each student was asked to identify and articulate what their goals were for the support they were accessing. Those individuals who met with a therapist collaboratively developed their goals through conversations with the *Kooth Student* therapists. These therapists were all trained to work online in a pluralistic, goal focused manner (Hanley et al., 2015). All goals inputted into the CoGS were then categorised by *Kooth Student* therapists using the taxonomy devised by Ersahin (2016). Each time the individual attended a session of therapy, they were asked to monitor any progress towards the completion of the goal, with each goal being assessed on a 10-point scale alongside the online therapists (0 = starting point; 10 = goal achieved). This process may also lead to individuals discarding goals and articulating new goals for their work with the therapist.

### Data Analysis

This paper provides a descriptive account of the goals that have been articulated and the subsequent movement towards achievement. In the first instance (and reported here), descriptive statistics were used to explore the type of goals articulated by the students. As noted above, these were coded using Ersahin’s taxonomy of goals (Ersahin, 2016) by the service staff. Secondly, the change reported through ongoing session-by-session monitoring is reported for everyone who took part in more than a single session of support.

## Results

This section provides an overview of the analysis of 12 months of routine evaluation data. It starts by reflecting upon demographic make-up of those who set goals during this period and what type of therapy they accessed. It then goes on to consider the types of goals set and the chronicled movement of these goals.

### Demographic Information and Session Type

**Figure 1:**
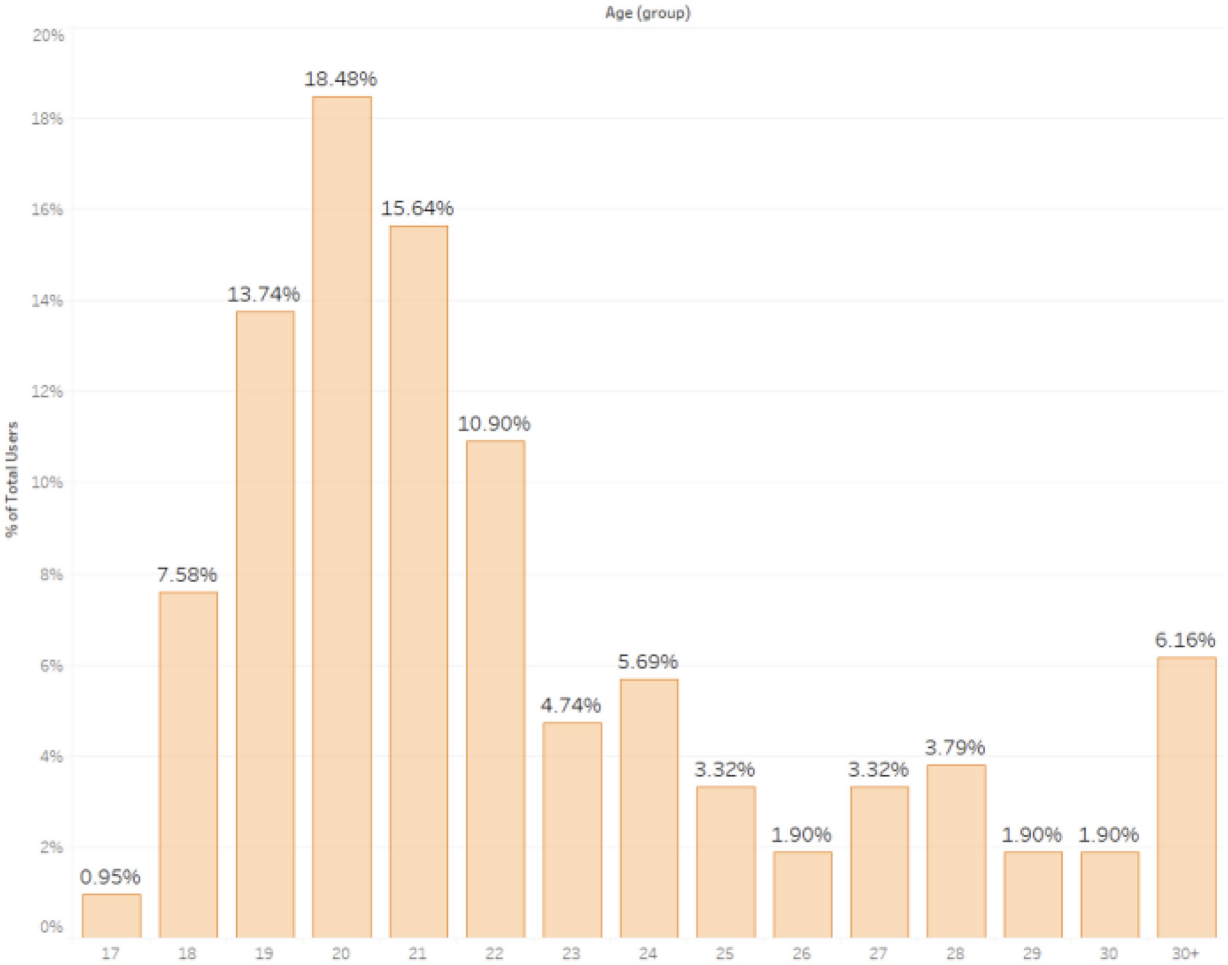
The age distribution of individuals who set goals in Kooth Student.

Figure 1 provides an overview of the individuals who set goals in the *Kooth Student* service. More than 50% of the student service users were aged between 18 and 22 (59%). 19% of users were male and 79% female. The remaining individuals identified as a gender or gender fluid. 17% of users were from a black, Asian or minority ethnic group. The referral source was predominantly through the university itself (27%) or the Internet (23%). Finally, the therapists chronicled the major presenting issues talked about in the sessions to be anxiety/stress (17%), depression (11%), self-worth (7%), confidence (7%), suicidal thoughts (4%), access to resources (4%), motivation (4%), family relationships (4%), exam stress and self-harm (3%).

**Figure 2:**
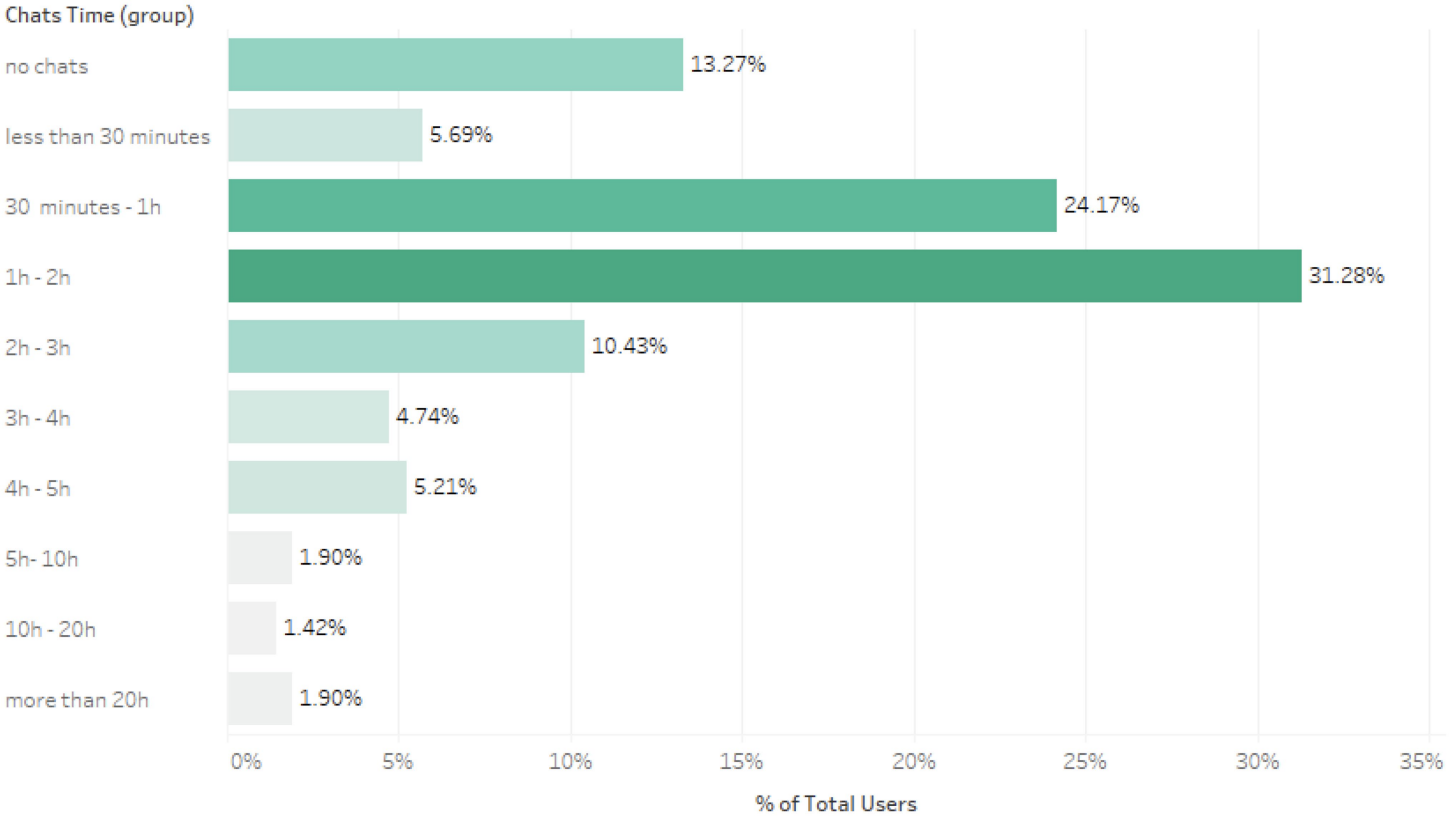
The time period each individual spent chatting to a therapist.

The individuals accessing therapy generally spent an average mean of 1 to 2 h accessing the services. Figure 2 provides an overview of the time individuals spent using the service. This time generally reflected individuals accessing multiple meetings, with structured pre-booked sessions tending to last 50 min while drop-in chats were often around 30 min in duration. 13% of the individuals who used the resource did not go on to meet with a therapist.

### The Goals That Students Set

**Figure 3:**
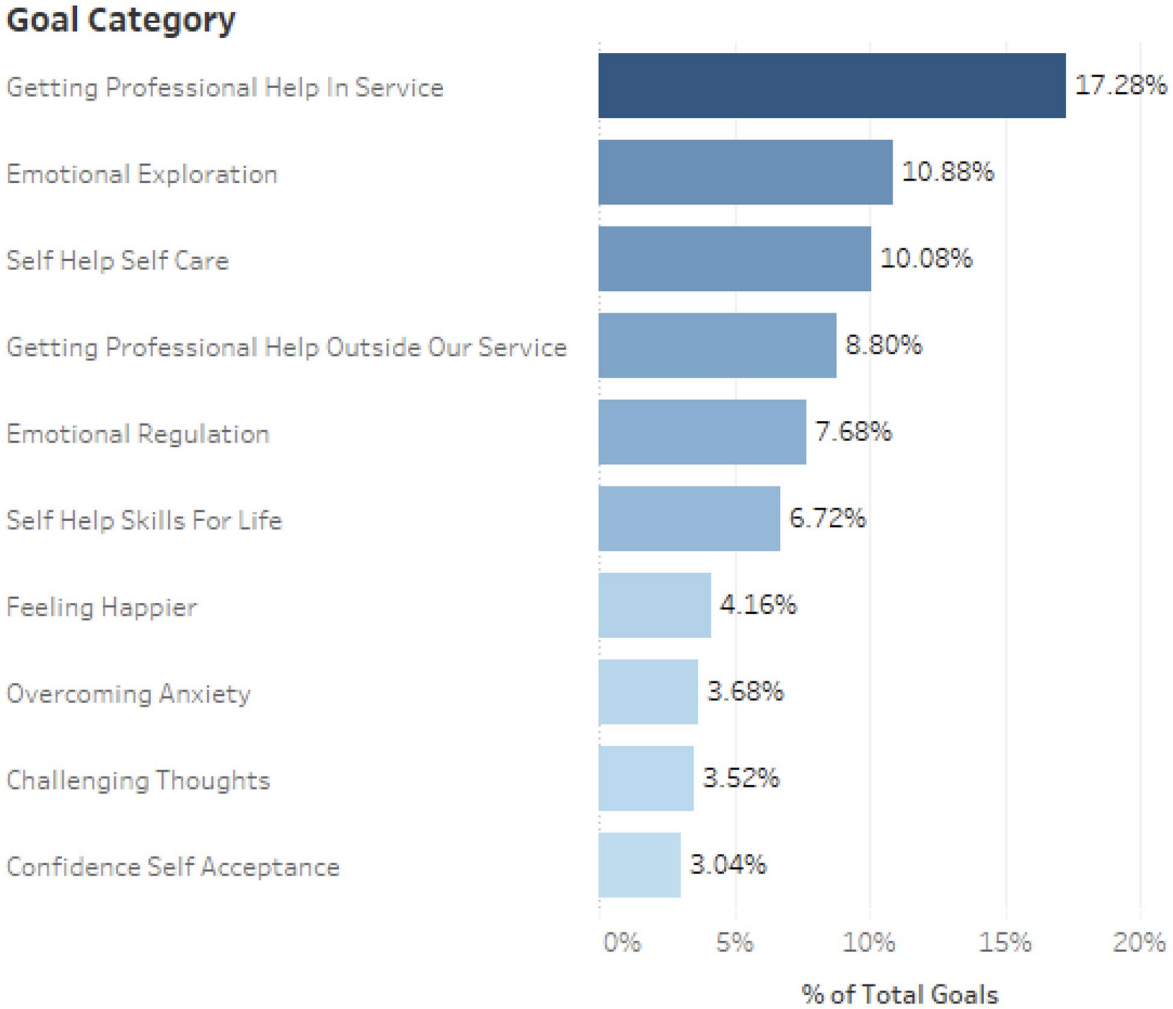
An overview of the top 10 goals set in Kooth Student.

The 211 students set a total of 625 goals, each setting a mean average of three goals. Figure 3 provides an overview of the top 10 goal categories that were created. As is evident here, getting professional help within the service proved the most common goal, with emotional exploration reflecting the second most common goal.

### Progress With Goals Set

Table 1 provides an overview of the goals articulated and the mean average movement towards achieving each of the goal types that had been set. Of the 625 goals set, 383 goals (articulated by 186 individuals) showed no movement during the time period in question—as these did not move, they are not considered in Table 1. A total of 242 goals (articulated by 73 individuals) did show movement towards achievement. The mean average movement of goals on the 10-point scale was 7.74. As is noted on the table, the goals associated with ‘Managing depression and Low mood’ and ‘Feeling happier’ were most successfully achieved, while the goals associated with ‘Challenging own behaviour’, ‘Grief’ and ‘Motivation’ moved least.

**Table 1:** Summary of the types of goals articulated and average movement of these goals during therapy.

Goal category	Goals	Users	Avg goals per user	% achieved	Goal movement
Managing depression or low mood	2	2	1.0	100.00	10.00
Feeling happier	6	6	1.0	100.00	10.00
Getting professional help outside our service	29	9	3.2	89.66	9.31
Suicidal thoughts	8	3	2.7	87.50	8.88
Getting professional help in service	55	30	1.8	81.82	8.98
Friendships	4	4	1.0	75.00	7.75
Emotional regulation	14	9	1.6	71.43	8.14
Confidence/self-acceptance	7	7	1.0	71.43	7.86
Self-harm	3	2	1.5	66.67	7.00
Enjoying self	7	5	1.4	57.14	6.14
Emotional exploration	30	24	1.3	56.67	7.17
Self-help skills for life	9	8	1.1	55.56	7.67
Overcoming anxiety	9	8	1.1	55.56	7.33
Speaking up/communicating better	4	4	1.0	50.00	7.50
Sleep issues	2	2	1.0	50.00	5.50
School/college/training	6	4	1.5	50.00	7.17
Career aspirational	2	2	1.0	50.00	5.50
Self-exploration	7	6	1.2	42.86	7.00
Self-help/self-care	17	14	1.2	41.18	6.00
Family relationships	3	3	1.0	33.33	5.33
Challenging thoughts	7	6	1.2	28.57	4.71
Getting more help from significant others	5	5	1.0	20.00	4.40
Motivation	1	1	1.0	0.00	5.00
Grief	1	1	1.0	0.00	2.00
Challenging own behaviour	4	3	1.3	0.00	5.25
Grand total	242	73	3.3	65.29	7.74

When comparing those who just created goals to those who actually moved towards achieving their goals, there was better engagement with goals from younger individuals (18–22), with 73% of this cohort showing movement in goals set. Further, it was also noted that males were less likely to set goals and less likely to move towards achieving them (15% of individuals), whereas females showed increased movement (84% of individuals).

## Discussion

This study set out to explore what type of goals students set within online therapy and whether they were achieving them or not. In this section, we will reflect briefly on each of the research questions in turn.

The individuals who used the online therapy service were predominantly young female students, 22 and under. Such findings prove similar to other studies examining service usage of therapy services for younger people, these appearing to show that therapy is more attractive to female students both in in-person services (Cooper, 2013) and web-based services (Sefi and Hanley, 2012). They also reflect those who may be classed as ‘digital natives’ (Prensky, 2001), notably individuals who have never lived in a world without the Internet, may find web-based services more appealing than others outside of this age range. The number of students from black, Asian or minority ethnic groups proved relatively modest. The 17% of students identifying in this way does however prove higher than the 10% prevalence reported in statutory Child and Adolescent Mental Health Services (Frith, 2017). Such an increase may be attributed to comforting elements such as the perceived safety provided by the anonymous communication allowed online (Hanley and Wyatt, 2021).

When considering the types of goals that individuals set, it is notable that these are wide ranging. These included goals that were classified as ‘intrapersonal goals’, interpersonal goals’ and ‘goals on self, related to others’. In contrast to goals set in similar services for younger age groups, the goals included less focusing upon getting help outside of the service itself (Hanley et al., 2017). Such a difference, may be due to the older age of the individuals accessing support, with these individuals potentially being more aware of services that are available, and making a choice to access support online. When considering the movement towards goal achievement, it is notable that practical goals that focused upon getting more help, both inside and outside the service, were most likely to be achieved. In contrast, self-help/self-care goals were less likely to be achieved. Such a trend appears understandable as the practical goals are likely to be more attainable in a short period of time, whereas achieving self-help/self-care goals are likely to need a longer period of time.

Where the articulated goals moved, it was notable that students reported high levels of goal achievement and that these goals moved further than in comparable data with young populations (Jacob et al., 2021). As with earlier studies in web-based therapeutic resources (Musiat et al., 2014; Santucci et al., 2014), despite aiming to be student focused, the service had a relatively high attrition rate. Only 73 of the 211 service users articulated goals that moved during the time they were engaged with the service. Such a process may demonstrate that individuals take the opportunity to explore the service, before engaging fully. Further, it is important to note that web-based services attract individuals that only use the service once (Chardon et al., 2011). *Kooth Student* proves no exception and, as a consequence, a number of the individuals who articulated goals would not have returned to them to report progress. In accounting for this, some studies only consider those individuals’ who return to the goals that they articulate (Jacob et al., 2021), whereas the data reported here included goal scores that both were and were not returned to. Given the varied ways that individuals use web-based services, the importance of developing appropriate means of successfully monitoring such work proves vitally important for service providers such as this.

## Strengths, Limitations and Future Research

This paper provides a novel summary of a large dataset generated over a 12-month period within everyday practice in a university therapy service. This is, to our knowledge, the first reflection of data exploring the use of goal-based outcomes within web-based therapy with a student population. While this paper presents the findings in a relatively neat and tidy way, it is important to acknowledge that routine evaluation data are commonly quite complex and uncontrolled. Such a phenomenon is potentially magnified online, with online technologies meaning that information is collated relatively easily and untangling the core elements can prove even more of a challenge (Sefi and Hanley, 2012).

This analysis did not consider the GbOM data alongside other outcomes captured by the service. Further investigation is needed to consider the interplay between idiographic and nomothetic outcome measures in this work. Further, the distance travelled data cannot tell us about single-session usage of such services. Evaluating single-session therapy needs further consideration to understand the reasons for such brief contacts and to assess its value. Additionally, more analysis is required to understand different user journeys in multifaceted online resources. To gain a richer understanding of these web-based mental health and wellbeing ecosystems, qualitative methodological approaches may prove more beneficial.

During the period, the data were collected in web-based therapy with this client group was in its infancy. Offering web-based therapies has however become increasingly commonplace as a consequence of the recent COVID-19 pandemic. Future research may utilise the findings here as a yardstick for comparisons before and after the pandemic. Such work will help to explore the way that therapy services have transformed and evolved during this unprecedented period.

## Data Availability Statement

The raw data supporting the conclusions of this article will be made available by the authors, without undue reservation.

## Ethics Statement

The studies involving human participants were reviewed and approved by University of Bolton Ethics Committee (Psychology). The clients/participants provided their online informed consent to participate in this study.

## Author Contributions

TH: lead author, conceptualization, methodology, data analysis, project administration, writing—original draft, and writing—review and editing. JP: conceptualization, methodology, and writing—review and editing. AS: conceptualization, data analysis and writing—review and editing. All authors contributed to the article and approved the submitted version.

## Conflict of Interest

AS is an employee of Kooth Plc.

The remaining authors declare that the research was conducted in the absence of any commercial or financial relationships that could be construed as a potential conflict of interest.

## Publisher’s Note

All claims expressed in this article are solely those of the authors and do not necessarily represent those of their affiliated organizations, or those of the publisher, the editors and the reviewers. Any product that may be evaluated in this article, or claim that may be made by its manufacturer, is not guaranteed or endorsed by the publisher.
